# Is Chloroquine Better than Artemisinin Combination Therapy as First Line Treatment in Adult Nigerians with Uncomplicated Malaria?-A Cost Effectiveness Analysis

**DOI:** 10.4314/ajid.v4i2.55145

**Published:** 2010

**Authors:** Shaibu O Bello, Aminu Chika, Aishatu Y Bello

**Affiliations:** 1 Department of Pharmacology, College of Health sciences, Usmanu Danfodiyo University, Sokoto, Nigeria; 2 Fertility Unit, Karaye Hospital, Emir Yahaya Road, Sokoto, Nigeria; 3 General Outpatient Department, Specialist Hospital, Sokoto

**Keywords:** Malaria, Artemisinin Combination Therapy, Chloroquine, Incremental Cost effectiveness, Quality adjusted life years, Nigeria

## Abstract

The current case management and drug policy of malaria in Nigeria recommended by the Federal ministry of health may not be appropriate for all age categories. This suspicion was tested by running a cost effectiveness analysis of two possible and alternative strategies: Artemisinin Combination Therapy (ACT) or Chloroquine and ACT only if CQ fails (CANACT), in adult non pregnant Nigerians aged 20–45yrs. The result confirms that ACT is indeed more effective but also more costly with an incremental cost effectiveness ratio (ICER) of #2,546,527.00 per QALY that is much higher than the estimated upper limit of #25,000.00 that either patients or provider may be willing to pay. The CANACT strategy may be the most cost effective strategy in this subgroup of Nigerian patients and also provides better value for money.

## Introduction

*Plasmadium falciparum* malaria is a potentially disastrous Infection, especially at the extremes of life (Gavazzi et al., 2004; WHO, 2008). In such age groups, effective and rapid control of parasitaemia may be life saving. Artemisinin and its derivatives are highly effective and rapid schizonticides and are reasonable as first line drugs for the treatment of malaria (de Vries and Dien ,1996; Hien and White, 1993). When Artemisinin is co-administered with a longer acting and efficacious anti-plasmodia drug with a different mechanism of action (a combination known as ACT), a high cure rate usually results (WHO, 2001). ACT is the recommended first line medication for the treatment of uncomplicated *Plasmadium falciparum* malaria, especially in areas where resistance to Chloroquine (CQ) and /or sulpadoxine-pyrimethamine is above 10% (WHO, 2006). Resistance to CQ in Nigeria is estimated to be between 25.8% (WHO, 2005) and 39.2% (Ukwuoma, 2005). On January 24, 2005, Nigeria dramatically banned CQ and Sulphadoxine-Pyrimethamine as first line drugs in the treatment of malaria in all age groups (Ukwuoma, 2005). It may be argued that the WHO recommendation has been hastily, perhaps canonically, adopted by Nigeria because evidence that robust cost-effectiveness studies was done as a guide to the adoption of ACT for all age groups of patients have not been presented. For an illness that is responsible for 60% of outpatient and cost the nation an estimated 132 billion Nigerian Naira (N) per year (Ukwuoma, 2005), changes in treatment policies need to be formally justified by careful consideration so as to optimize cost versus effectiveness. In Plasmodium malaria, it is plausible that the cost-effective and therefore the best treatment strategy may be different between age groups because age is known to modify diseases and treatment outcomes (Bello et al., 2005). The high morbidity and mortality associated with malaria in persons aged under 5 years and over 55 years is probably compelling argument for the use of ACT in these age groups. This argument may also be true in pregnant adults, epidemic malaria or in non immune persons but the same argument may not stand scrutiny in holo-endemic regions when applied to non pregnant adults in the age range 20–45 yrs (adult Nigerians or AN) who also constitutes about 56% of malaria cases (Bello et al., 2005). In this group, a case may be made for CQ as a first line drug irrespective of the current level of resistance because six to seven of every ten of patients should respond to CQ while 3–4 will be expected to fail and are the ones who really need the ACT. Furthermore, the probability of treatment success or failure to CQ and therefore the need to add-on ACT may be determined within 24–48 hrs of starting treatment because, when effective, CQ causes fever lyses in this time range (Shapiro and Goldberg, 2001; Mengesha and Makonnen, 1999). One of the expected consequences of treatment failure in AN is a longer duration of malaria morbidity because, by adult age, herd immunity is usually well established and mortality is extremely rare (Gavazzi et al., 2004). Other considerations that may guide decision is that while ACT has high rates of substandard preparations that contain between 74%and 77% (Atemnkeng et al., 2007, Raufu, 2002) of the value stated by the manufacturer and currently have treatment failures of about 1–18% (Ittarat et al., 2003; Ajayi et al., 2008a), CQ has substandard preparations that contain 0–87% (Ofonaike et al., 2007) but those that contain above 50% (89% of substandard CQ) of the label value are still efficacious in non-resistant parasite because CQ concentrates within parasite vacuoles (Shapiro and Goldberg, 2001). Two different strategies may therefore be considered to be available as potential first line treatments in adults with uncomplicated malaria namely-ACT or CQ plus ACT when needed (CQ plus as needed Artemisinin combination treatment-CANACT). The optimal of these treatment options may be identified after formal evaluation by a cost-effectiveness study. This study was conducted for this reason.

## Methodology

### The Model

A decision tree based on the model of Goodman et al (2001) but with modified terminal outcomes was developed using the software Treeage PRO^(R)^ 2008 (TreeAge Software, Inc., Williams, MA). A state transition (Markov) model was then developed using the same software. The intention of the model was to simulate the cost-effectiveness of a strategy that involves CANACT with ACT. It was assumed that all initial treatment would be outpatient. A Markov cycle length of 3 days was used because it was expected that based on clinical response, the next decision in the treatment chain should be made in 72hrs. Cure was defined as Adequate clinical response (ACR) using the WHO criteria (WHO, 2003) while failure was defined as absence of ACR. Parasitological response was not factored in because a state transition of 3 days is inconsistent with parasitological success that requires at least 28days to declare (WHO/CTD, 1996). Furthermore, anecdotal evidence suggests that day 28 parasitological screening is not routine in Nigeria and available evidence suggests that microscopy is only routinely used in about 21.6% of cases in non research settings (Njuguna and Qader, 2007). Quinine was considered as the final treatment option of interest after ACT and therefore the decision pathway was terminated after Quinine failure and this was termed worst case scenario (‘Worstcase’) which could mean death, long term disability or long term treatment until either of these occurs or cure ensues. The probabilities at chance nodes were allocated from published estimates except that it was assumed that the chance of failure of ACT as first line treatment would be 10% as a consensus between the published clinical failure rate of 15–18% in observational studies (Ittarat et al., 2003) and the 1–2% failure rates reported in more formal randomized studies (Martensson et al.,2005; Meremikwu et al., 2006; Ajayi et al., 2008b). On the other hand, the chance of failure of ACT after treatment with CQ was accepted to be the frequently quoted 2% because ACT has been reported to be more effective against CQ resistant than CQ sensitive *Plasmadium falciparum* (Shapiro and Goldberg, 2001). It was estimated that severe malaria has equal chance of being treated outpatient or in patient because which of these events occurs depends on the willingness of the patient to seek secondary or tertiary level care and the availability of such level of care. It is therefore reasonable to expect the chances to be balanced (i.e. 50:50) in the Nigerian setting. Inpatient Quinine therapy was defined as Quinine plus all other drugs that may be co-administered (e.g. Intravenous infusions, Doxicycline etc.) and was therefore allocated a 1% chance of failure. Because the allocated nodal probabilities are published estimates and unbiased between ACT and CANACT, it was expected that minor errors may not influence outcome. However, it was pre-specified to perform sensitivity analysis using the various ranges of published estimates of treatment outcomes to test if errors in such estimates may affect the results. We tested the outcome between Chloroquine failure rates of 10 % to 90 % at 5% intervals. We also tested the outcome between ACT failure rates of 1% to 20 % at 5% intervals.

### Cost

This analysis is expected to provide additional guide for clinical and policy decisions. We, therefore, first considered cost and effectiveness from the providers' perspective. We recognize that only 38% of Nigerian patronize government facilities for malaria care (Ejezie et al., 1990), therefore CE from the consumer's perspective was also evaluated. In ideal set ups, doctors, nurses, supplies and administrative costs are defined, however cost data are extremely hard to estimate in developing economies (Onoka et al., 2007). We therefore used a simple imputation costing system (Johnston et al., 1999) by allocating #15000.00 for these cost per cycle of inpatient admission (which we defined as every 3 days) and used simple market survey to quantify the real cost of drugs. All costs were estimated in Nigerian Naira (#) because the study is focused on treatment in Nigeria and conclusions are expected to be valid only for the treatment of malaria in Nigeria. The exchange rate on the date costing was documented for this study was #154.1 for $1.00 or 0.696 Euros (Worldmate, 2009)

### Effectiveness

In the decision tree and Markov model, effectiveness was measured as Quality-Adjusted Life Years (QALYs) which provides a numerical measure of health over time and may be estimated as the product of the quantity of life gained per treatment episode and the quality. QALY was estimated from both the provider's and the consumer's perspectives. From published data, an adult Nigerian may develop malaria two times per year (Olanrewaju and Johnson, 2001). The Quantity of life gained per treatment was therefore estimated as 6 months. Age weighing and discounting were not done because these are only important when Disability Adjusted Life Years (DALY) is used as the index of effectiveness (Myamoto and Eraker, 1985; Sassi, 2006). Quality of life was estimated from the patients' perspective. ‘Cure’ was therefore allocated a score of 1 and ‘Worstcase’ a score of zero because death is a possible outcome. Quality score for severe malaria in Nigerian was taken as 0.3 (Goodman et al., 2001). Quality score for uncomplicated malaria was estimated to be 0.67±0.26 as determined by a pilot study using the visual analogue scale (VAS) on 10 randomly selected male and female Nigerian and asking respondents to allocate on a visual scale of 0–100 their estimate of the quality of life when with malaria against when fully healthy. Person trade-Off (PTO) method was not used because we agree with the criticism that values allocated by expert panels may not represent values of others (i.e. is not patient oriented) and that PTO includes forced consistency between questions that are quite different (Bleichrodt and Johannesson, 1996; Arnesen and Nord, 1999). The mean score was used in the models while the standard deviation was used in the sensitivity analysis. We compared cost effectiveness by using Incremental Cost Effectiveness Ratios (ICER).

### Willingness to pay

We used the internationally accepted $50,000 ICER threshold (Hirth et al., 2000) for sensitivity analysis but used #25000 as the conservative WTP by the provider. This was estimated from the per capita income of Nigeria which stood at $2300 in 2008 (Tong, 2008) adjusted by the percentage allocated to the health sector in the 2008 and 2009 Nigerian Budget (The Federal Government of Nigeria, 2009). Also, Jimoh et al (2007) have shown that Nigerians are willing to pay (WTP) # 1,112 monthly (i.e. #13344 every year) for the treatment of adult malaria. We therefore used this as the WTP for one QALY Gained from the consumers' perspective. We captured all three thresholds in our analysis in order to address the uncertainties of policy changes.

## Results

**Figure 1:**
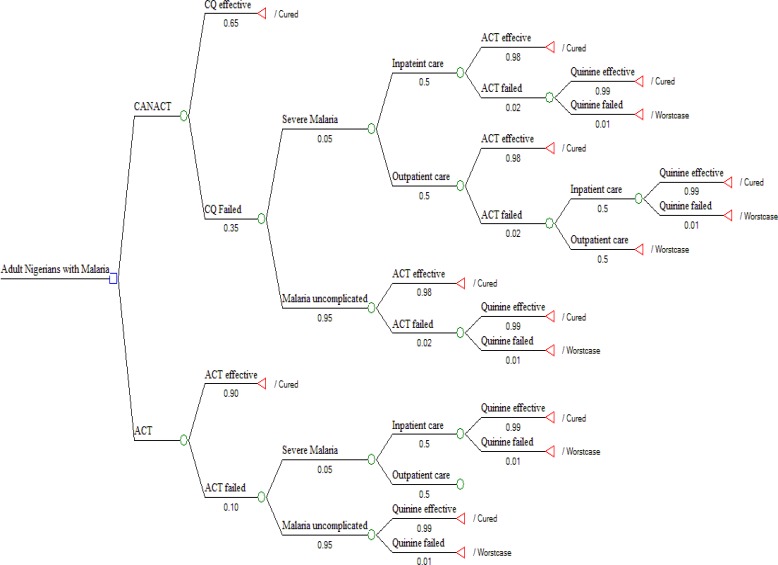
Decision tree used to compare the cost-effectiveness of Artemisinin-based combination therapies (ACTs) versus CQ plus as needed ACTs (CANACT). 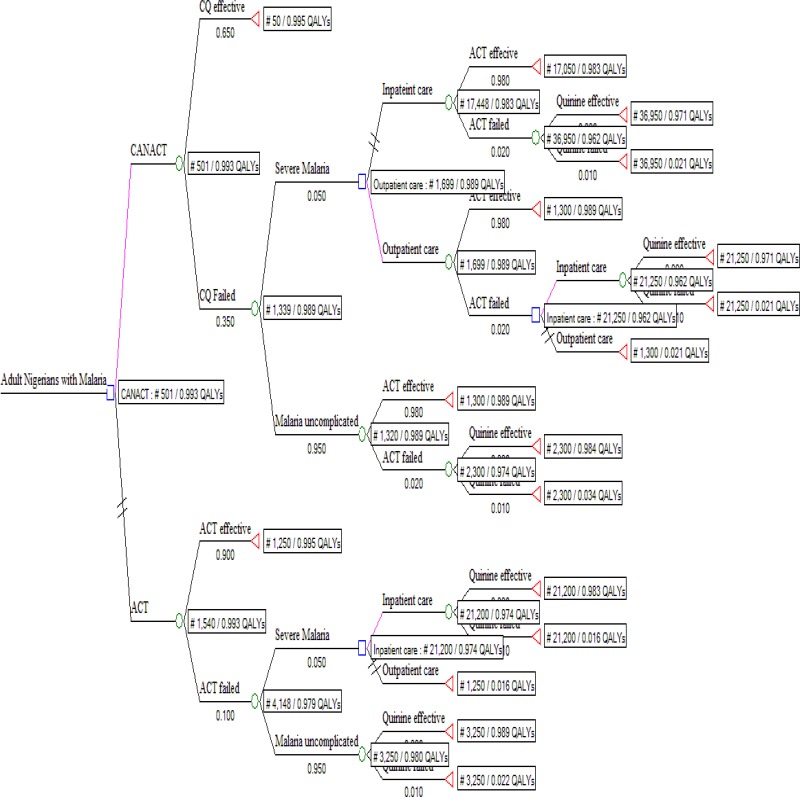

**Figure 2:**
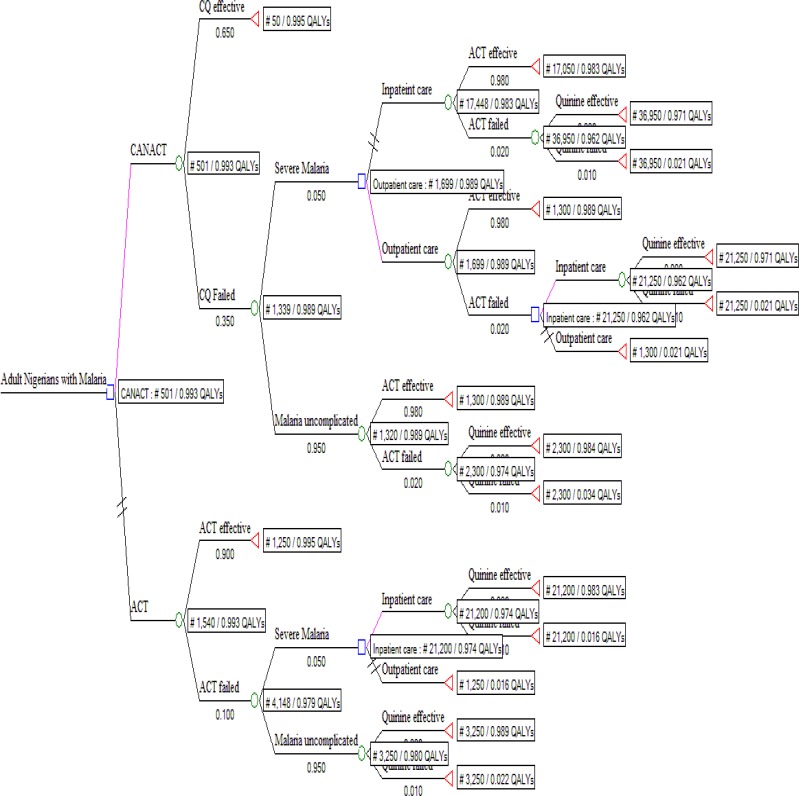
Rolled back decision tree with best pathway analysis When the decision tree was rolled back, Treeage 2008^(R)^ picked CANACT as the optimal pathway. This outcome did not change even at a willingness to pay #200000 (i.e. when cost was absolutely not an issue). 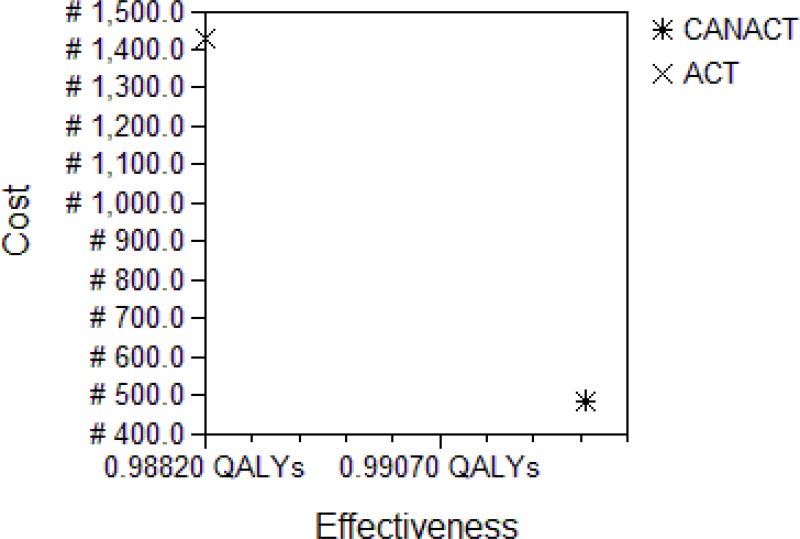 //= crossed out or unfavourable pathway

**Figure 3:**
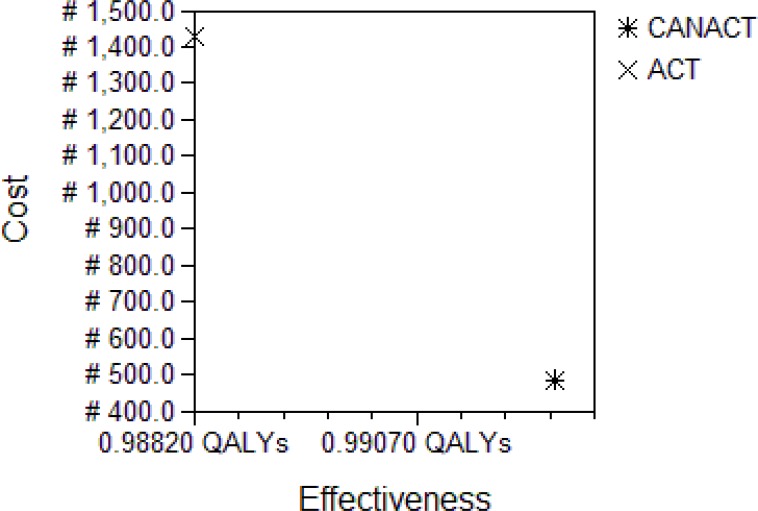
Cost-Effectiveness analysis Plot at WTP of #1150 The analysis was taken at the decision point “Adult Nigerians with Malaria”. The ACT strategy is obviously dominated by CANACT. The graph is from the consumer's perspective and captures the current program where adult Nigerians pay for their own treatment. The WTP of #1150 by Nigerians for the treatment of malaria reflects the findings of Jimoh et al (2007).

**Figure 4:**
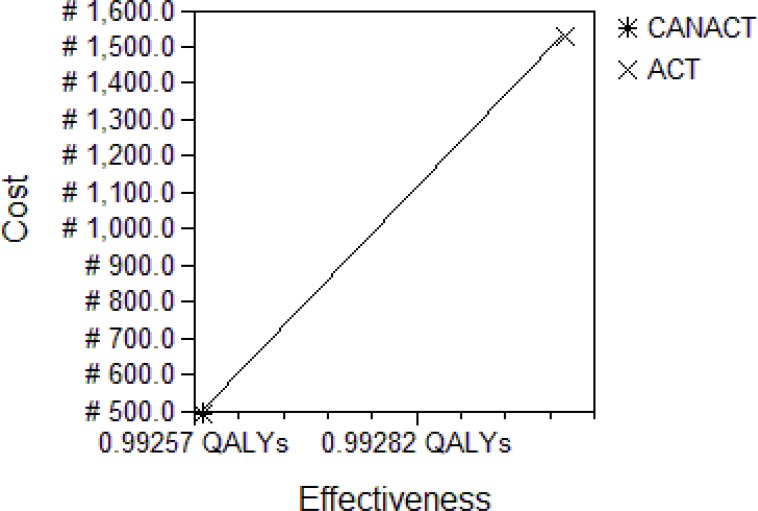
Cost-Effectiveness analysis Plot at WTP #25,000.00 – #7,705,000.00 The analysis was taken at the decision point “Adult Nigerians with Malaria”. This plot captures a government (provider) sponsored program. ACT is then more effective but also much more costly than CANACT. This relationship remains the same between WTP of #25,000.00 to #7,705,000.00 (when cost implication is considered to be of little consideration).

The decision tree, Figure 1, followed the possible outcome of an AN with uncomplicated malaria treated with either CANACT or ACT. The cost of the items in the pathway is as listed in Table 1. The probability estimates of all components of the tree are as listed in Table 2. CANACT had a CE of #501.1 per 0.99258 QALY while that of ACT was #1,539.8 per 0.99299 QALY. The ICER of ACT was #2,546,527.00. Other CE outcomes are as listed in Table 3. From the calculated (rolled back) tree (Figure 2),if the CANACT strategy is chosen, when CQ fails, the best pathway (most CE) pathway then becomes outpatient rather than inpatient ACT irrespective of whether the malaria remain uncomplicated or severe ( CE of #1699 per 0.989 QALY for outpatient care compared to #17448 per 0.983 QALY for inpatient care) but when ACT fails, inpatient quinine(CE #21200 per 0.974 QALY) is more CE than outpatient quinine(CE #1250 per 0.016 QALY ) in severe malaria while outpatient quinine is more CE if the malaria remain uncomplicated. The optimal pathway after ACT remained the same irrespective of whether it was used as first line or backup (second line) medication. The result of testing the uncertainties in the failure rates of CQ and ACT are shown in Table 4. From the Monte Carlo simulations, if these strategies are used now and maintained for the next 10 years, CANACT dominates ACT at WTP of #1150.00 (Figure 3) to #20,000.00 but no strategy is dominated between WTP of #21000 and #7,705,000.00 (Figure 4). The net health benefit of the ACT strategy is never superior to the CANACT strategy at all levels of WTP (Figure 5a). At a threshold willingness-to-pay of #25 000 per QALY, 75% of resources will be optimally utilized with the CANACT strategy compared to 22% with the ACT strategy (Figure 5b). From the Markov's model, when all possible outcomes are considered given the available data at a WTP of #25000, the ACT strategy is either less effective and less costly or less effective and more costly 65% of the time when compared to the CANACT strategy (Figure 6).

**Table 1:** Estimates of cost used for the decision tree

Item	Cost per unit(#)	Source
**Quinine Tablet**	#25.00	Market survey by BSO and HA
**Quinine Injection**	#50.00	Market survey by BSO and HA
**Chloroquine Tablet**	#5.00	Market survey by BSO and HA
**Chloroquine Injection**	#15.00	Market survey by BSO and HA
**Artheter Injection**	#250.00	Market survey by BSO and HA
**Artesunate Injection**	#350.00	Market survey by BSO and HA
**Needles & syringes**	#10.00	Market survey by BSO and HA
**Intravenous fluid per 500mls**	#150.00	Market survey by BSO and HA
**Giving set**	#25.00	Market survey by BSO and HA
**Plaster**	#60.00	Market survey by BSO and HA
**Cannula**	#60.00	Market survey by BSO and HA
**Inpatient Quinine care (including** **fluids , Doxicycline)**	#4925.00	Hospital survey by BSO and AYB
**Non drug cost of inpatient care**	#15000.00	Best anecdotal estimate by BSO, CA and AYB.

BSO=Bello Shaibu Oricha, CA=Chika Aminu, AYB=Aishatu Yahaya Bello, HA=Hauwa Abdullahi

**Table 2:** Estimates of probabilities used for the decision tree

Input variable	Type of probability distribution	Best estimate in Nigeria(%) and source	Lowest estimate in Nigeria (%) and source	Highest probable estimate anywhere (%) and source	Estimate used for tree (%)
**Risk of CQ** **failure**	Point estimate	38.0; Ogungbamigbe et al., 2008	13.4; Olarewaju, 2001; Ekanem et al., 2000	88.0; WHO 2000; WHO 2008.	35.0
**Risk of ACT** **failure**	Point estimate	2.0; Meremikwu et al., 2006	1.0; Ajayi et al., 2008	18.0; Ittarat et al.,2003	10.0
**Risk of Quinine** **failure**	Point estimate	2.0; PrayGod et al.,2008	14.7; Adagu et al.,1995	21.0; Yeka et al.,2009	10.0
**Risk of ACT** **failure in CQ** **resistant malaria**	Point estimate	2.0; Meremikwu et al., 2006	1.0; Ajayi et al., 2008	2.0 Meremikwu et al., 2006	2.0
**Risk of severe** **malaria in AN**	Anecdotal estimate	1.0; Snow et al,1999	1.0; Snow et al,1999	5.0; Makani et al., 2003.	5.0
**Risk of death** **from** **U/MDRSPfm in** **AN**	Point estimate	NA	NA	100.0 ; WHO,2000	99.0

NA=Not available, AN = Adult Nigerians, U/MDRpfm= Untreated/multidrug resistant severe *Plasmadium falciparum* malaria.

**Table 3:** CE table of CANACT versus ACT

Strategy	Cost (#)	Incremental Cost	Effectiveness (QALY)	Incremental Effectiveness	Cost-Effectiveness (#/QALY)	Incremental Cost-Effectiveness ratio(#/QALY)
**CANACT**	501.1		0.99258		505.00	
**ACT**	1,539.8	1,038.6	0.99299	0.00041	1551.00	2,546,527.00

**Table 4:** Sensitivity analysis at various levels of predicted Artemisinin and Chloroquine resistance.

Level of resistance to Chloroquine (%)	Level of resistance to ACT (%)	Cost -effectiveness of ACT strategy (#/QALY)	Cost-Effectiveness of CANACT strategy (#/QALY)	Optimal Strategy
**10**	10	1540/0.993	179/0.994	CANACT
**20**	10	1540/0.993	303/0.993	CANACT
**30**	10	1540/0.993	437/0.993	CANACT
**40**	10	1540/0.993	566/0.992	CANACT
**50**	10	1540/0.993	694/0/992	CANACT
**60**	10	1540/0.993	823/0.991	CANACT
**70**	10	1540/0.993	952/0.991	CANACT
**75**	10	1540/0.993	1017/0.990	CANACT
**80**	10	1540/0.993	1081/0.990	ACT
**85**	10	1540/0.993	1146/0.990	ACT
**75**	0	1250/0.995	1017/0.990	ACT
**70**	0	1250/0.995	952/0.991	CANACT

This sensitivity analysis presented in this table was done at WTP of #200,000 –#7,705,000.00

**Figure 5a:**
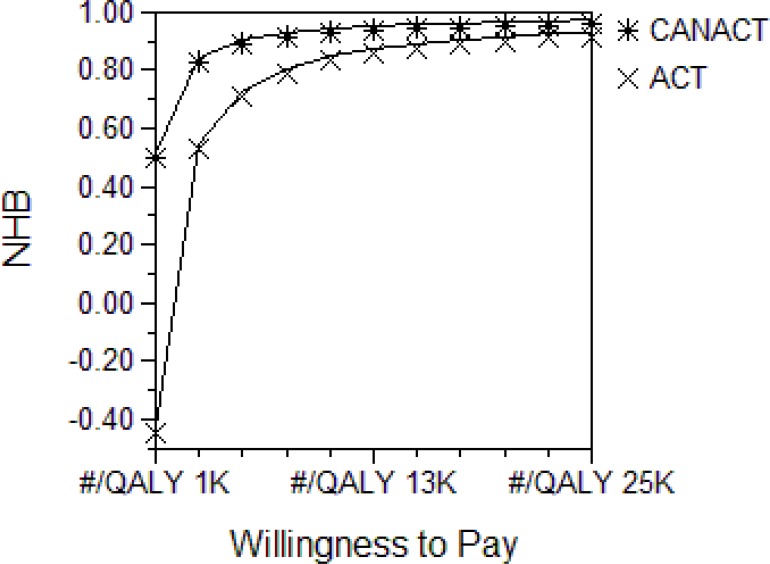
Net Health Benefit (NHB) and willingness -to- pay The graph displays the NHB (effectiveness -Cost/ WTP) of each strategy at various levels of WTP. NHB describe the uncertainties in CE models and may specify the WTP at which a given strategy becomes more CE than the comparator. The most CE comparator is the one with the highest NHB at a given WTP. In this graph, the NHB of the ACT strategy is either inferior or equal to that of the CANACT strategy at all levels of WTP examined (up to #7,705,000.00). The plots have been truncated at #25000 because the two lines are equal beyond a WTP of #25000.

**Figure 5b:**
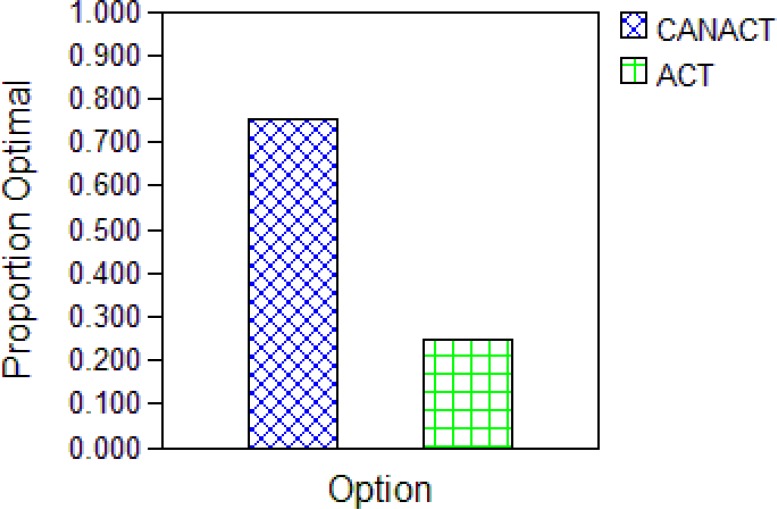
Net health benefit optimality graph based on a Willingness-to-pay of #25000 The graph displays against each option (strategy) the percentage of 1000 Monte Carlos Micro stimulation in which that option had the highest net health benefit. CANACT had 75% optimal benefit and ACT had 22%. More value for money is obtained from the CANACT than the ACT strategy.

**Figure 6:**
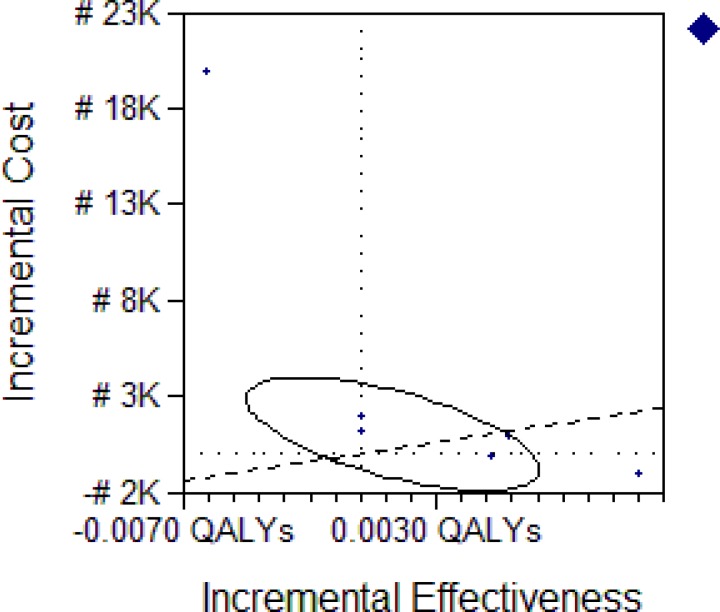
ICE scatter plot of ACT Versus CANACT strategy

The From the simulation statistics, ACT is more costly and more effective than CANACT at WTP of #25000. Following standard protocol (Fenwick et al., 2004), CANACT was therefore used as the baseline strategy and ACT as the comparator. The ellipse describes the probability that ACT is cost effective compared to CANACT (the origin) given the available data at the specified WTP. WTP line in the graph intersects points having the specified ICER value, and the region below the line includes cost-effective points. The ellipse falls within all of the quadrants, with 31% contained within the NW quadrant (i.e. dominated) and 28% within NE quadrant (i.e. more effective and more costly) and with only 35% cost effective (below the WTP slanting line). This plot makes a strong case for the baseline strategy (CANACT).

## Discussion

This study has confirmed that the ACT strategy is indeed more effective in adult non pregnant Nigerians than the CANACT strategy but that it is also much more costly; with an ICER of #2,546,527.00 per QALY gained. This ICER is also much below the internationally accepted ICER limit of $50,000.00 (or #7,705,000.00 at the current exchange rate of #154.1 per $). This study, therefore, appears to support the adoption of ACT as the first line strategy by the Nigerian Government. A careful review of the result using contemporary pharmacoeconomic principles may suggest otherwise. A number of commentators have suggested that the goal of the health intervention is to optimize health gain for limited funds (Claxton et al., 2002) and that, given this goal, it is the likely value of an intervention that should guide decision (Claxton et al., 2002; O'Hagan et al., 2000). A continuing argument is on how much value we may put to health. The United States of America (USA)'s ICER threshold of $50,000.00 per QALY as ideal has been used as the standard WTP internationally (Hirth et al., 2000) but this has been criticized as flawed (Ubel et al., 2003). For this reason, various countries and organizations have used widely different and less ambitious thresholds (Devlin and Parkin, 2004; George et al., 2001; Hoch, 2004). If the provider is willing to pay the standard ICER threshold, then ACT strategy is the best strategy. This study has revealed than when consumers have to pay for care and the government is only WTP a fraction of the standard ICER, the CANACT strategy dominates ACT at the current level of CQ resistance. Also, the CANACT strategy remains dominant until either the background resistance to CQ is at or above 75% and the failure rate of ACT is zero or the failure rate of ACT is at or above 10% and the failure rate of CQ is 80%. This study has provided strong evidence that even if the provider was willing to pay the standard ICER threshold more value for money is obtained with the CANACT strategy at the current CQ failure rates because both the optimality analysis and NHB favours it..

It may be argued that irrespective of policy statements, whether the policy is ideal for a situation is best evaluated from reality. Our result has presented that given the WTP of the Nigerian public for malaria as shown by Jimoh et al. (2007), the ACT strategy is the wrong strategy for AN. This is may be one of the reasons for the poor compliance to the ACT policy by physicians and patients (Ajayi et al., 2008b; Bosman and Kamini, 2007). If policies are not supported financially, it is probably more likely that the interplay of the payers' value versus ability dictates which policy is executed.

The optimal pathway shown in the rolled back decision tree further illuminates the role of CE in guiding even treatment algorithms, given that resources are restricted. Again, the pathway that is chosen depends on the WTP. Developed nations may disregard cost values below #7,705,000.00 per QALY and the WHO may be right to have recommended policies to countries based on the non-inferiority principle, especially when such policies are well funded and supported ex-government, but this study has shown that adoption by less developed economies is best based on pharmaco-economic data.

## Conclusion

Given the current levels of treatment failures of CQ, both CANACT and ACT strategy remain CE treatment strategies in AN with uncomplicated malaria. The banning of CQ by the Nigerian government was probably hasty and is not supported by the current level of government funds available for malaria control. In AN, the CANACT strategy gives more value for money and may be enhanced by adequate control of counterfeit medications. It may be important to conduct further studies to broadly define the optimal strategy for other age categories as guide to resource optimization.
